# A Novel Algorithm to Improve Digital Chaotic Sequence Complexity through CCEMD and PE

**DOI:** 10.3390/e20040295

**Published:** 2018-04-18

**Authors:** Chunlei Fan, Zhigang Xie, Qun Ding

**Affiliations:** 1 Electrical Engineering College, Heilongjiang University, Harbin 150080, China; 2 Department of Electronic and Information Engineering, Polytechnic University, Hong Kong 999077, China

**Keywords:** chaotic system, empirical mode decomposition, permutation entropy, image encryption

## Abstract

In this paper, a three-dimensional chaotic system with a hidden attractor is introduced. The complex dynamic behaviors of the system are analyzed with a Poincaré cross section, and the equilibria and initial value sensitivity are analyzed by the method of numerical simulation. Further, we designed a new algorithm based on complementary ensemble empirical mode decomposition (CEEMD) and permutation entropy (PE) that can effectively enhance digital chaotic sequence complexity. In addition, an image encryption experiment was performed with post-processing of the chaotic binary sequences by the new algorithm. The experimental results show good performance of the chaotic binary sequence.

## 1. Introduction

With the rapid development of computer technology and network communication technology, information has become an important asset in today’s society. Therefore, the confidentiality of personal information has become more and more essential. For example, internet data transmission and confidential phone and bank cards require adequate security and confidentiality measures. Therefore, the study of secret communication and cryptography has become an urgent issue. At present, the chaotic signal has benefits such as intrinsic stochasticity, initial value sensitivity, and synchronizing characteristics. Therefore, some traditional chaotic systems with a self-excited attractor are widely used in secret communication and have significant advantages [1,2,3,4,5]. Further, in recent years a hidden chaos attractor has been found, which makes the development of a high-dimensional nonlinear system an attractive challenge [6,7,8,9]. At present, most scholars primarily study the dynamic characteristics of hidden attractors. In this paper, we aimed to study chaos with a hidden attractor from the perspective of secure communication and cryptography. Chaos with a hidden attractor is used as a digital chaotic sequence generator with the purpose of encrypting private data. However, in the process of quantization, calculation precision is a crucial factor that degenerates the dynamic characteristics of a chaotic system so that the complexity of a digital chaotic sequence does not satisfy the requirements of information security and cryptography [10,11]. Aiming to solve this problem, Du [12] put forward an algorithm to improve the performance of chaotic binary sequences based on Karhunen–Loève (K–L) transformation. Zhou [13] proposed to scramble the chaotic binary sequence by m sequence in order to improve the complexity of the digital chaotic sequence. Cernak [14] came up with a method to improve the randomness and periodic length of the chaotic binary sequence by perturbing parameters of the chaotic system. Based on the above analysis, these algorithms improve the performance and complexity of digital chaotic sequences by reconstructing the binary sequence method. In this paper, we attempted to generate high complexity in the chaotic sequence based on digital signal processing technology.

Empirical mode decomposition (EMD) in digital signal processing has been extensively applied in nonlinear signal processing [15,16,17,18]. EMD was first proposed by Huang et al. [19,20,21]. It is an effective tool for analyzing nonlinear and non-stationary signals. The EMD method is closely related to the corresponding Hilbert transform method. Through the decomposition of nonlinear and non-stationary signals, a series of intrinsic mode functions (IMFs) are obtained, which makes each IMF a stable signal for narrowband [22]. The IMFs play a crucial role in the analysis of non-stationary or nonlinear signals. However, there are some problems with the EMD method, of which the main one is mode mixing. Complementary ensemble empirical mode decomposition (CEEMD) can effectively restrain the mode mixing of EMD at a certain level [23,24,25]. Based on the above considerations, we proposed a new algorithm which combines CEEMD with permutation entropy (PE) [26] to effectively improve the complexity of the digital chaotic sequence.

The rest of this paper is organized as follows: Section 2 describes a hidden chaos attractor with no equilibria. The dynamic characteristics of a complex chaotic system are studied by means of numerical simulation and theoretical analysis. Section 3 proposes a new algorithm to improve the complexity of the digital chaotic sequence. Section 4 considers image encryption with post-processing of the chaotic binary sequences by the algorithm outlined in Section 3. The security of the encrypted image is analyzed through key sensitivity, information entropy, and histogram analysis. Section 5 summarizes the discussions of this paper.

## 2. The Characteristic Analysis of a Chaotic System

**Figure 1:**
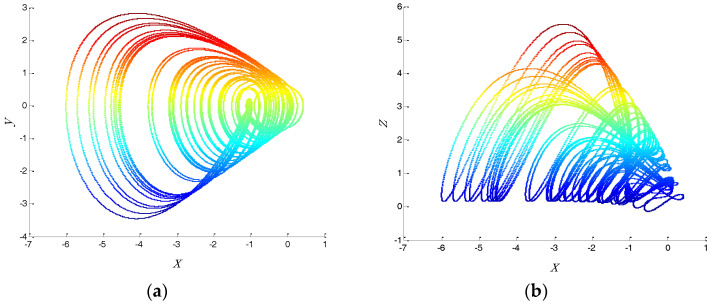

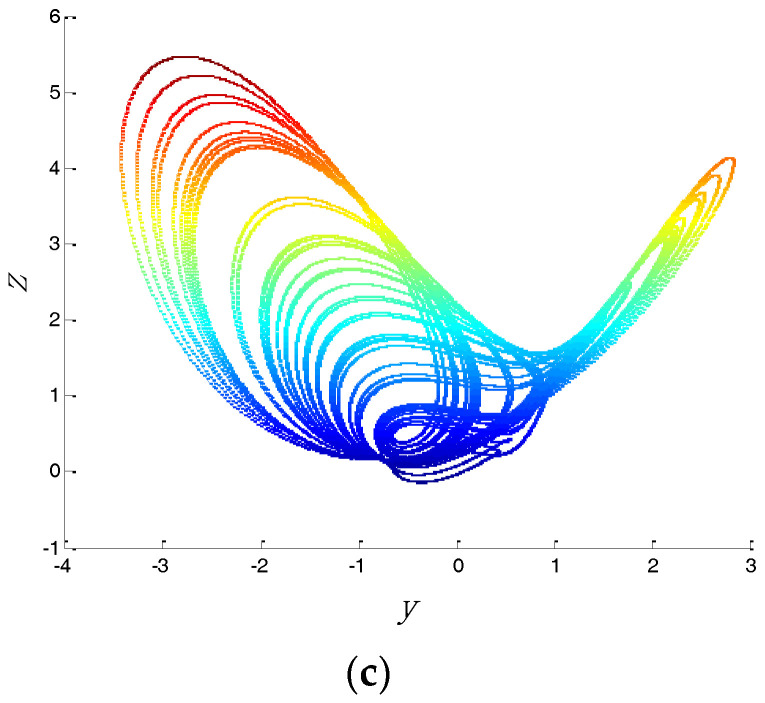
The different projections of chaotic attractor with: (**a**) *x*-*y*; (**b**) *x*-*z*; (**c**) *y*-*z*.

In this section, a system can be expressed as the following set of differential equations:

(1) $$\{\begin{array}{c} \dot{x}=-y\\ \dot{y}=cx+z\\ \dot{z}=ay^{2}+xz-b \end{array}$$

where a, b, c are real parameters. When a=2, b=0.35, c=1 and the initial value is (−1.6, 0.82, 1.9), the system displays a single-scroll chaotic system [27]. Different projections of the chaotic attractor for this system are shown in Figure 1.

Equilibria of the chaotic system can be obtained by solving $\dot{x}=\dot{y}=\dot{z}=0$. The equation is shown as follows:

(2) $$\begin{array}{ccc} \{\begin{array}{c} \dot{x}=0\\ \dot{y}=0\\ \dot{z}=0 \end{array} & \to & \{\begin{array}{c} 0=-y\\ 0=cx+z\\ 0=ay^{2}+xz-b \end{array} \end{array},$$

**Figure 2:**
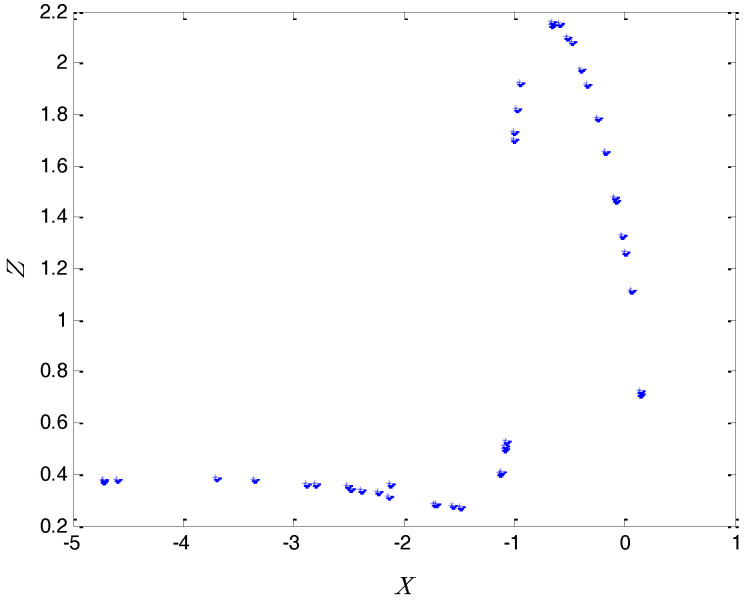
Poincaré map in the *x*-*z* plane.

**Figure 3:**
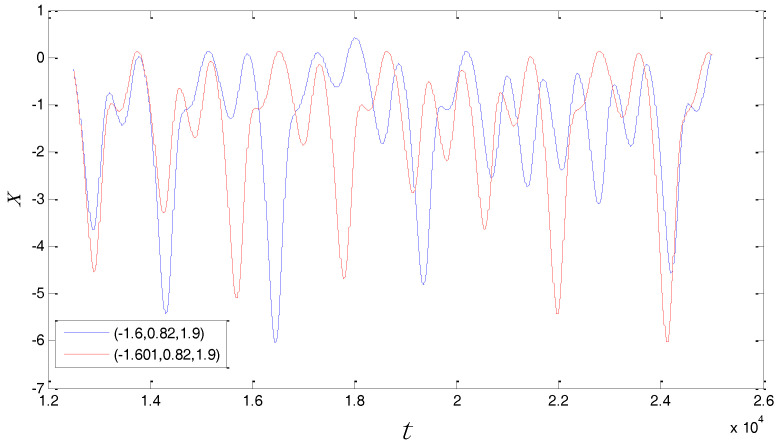
Initial value sensitivity for the time series x with the initial values (−1.6, 0.82, 1.9) and (−1.601, 0.82, 1.9).

However, it is easy to see in the chaotic system that when a=2, b=0.35, c=1, Equation (2) has no solution. Therefore, the chaotic system has no equilibria in this case. For the classification of chaotic attractors, if the basin of chaotic attraction intersects with any open neighborhood of an equilibrium, this attractor is called a self-excited attractor. However, if the basin of chaotic attraction is not connected with any equilibrium, this attractor is called a hidden attractor [28,29,30]. Therefore, the above chaotic system displays a hidden attractor in this case because it is a system with no equilibria. In addition, the Poincaré map of the system can be obtained in the $P=\{y=0|(x,z)\in R^{2}\}$ plane. For the above three-dimensional chaotic system $(x,y,z)\in R^{3}$, all (x,0,z) points were calculated by a MATLAB (R2012a, MathWorks, Natick, MA, USA) numerical simulation to obtain the Poincaré map. The Poincaré cross section projected in *x*-*z* is shown in Figure 2. The Poincaré cross section indicates that the system is a chaotic system through some dense points. Further, for the above chaotic system, the maximal Lyapunov exponent was calculated by a MATLAB numerical simulation. The maximal Lyapunov exponent can indicate the degree of the average divergence of the chaotic trajectory. If the exponent is more than zero, it denotes that the system has the sensitivity of the initial value. According to the result of the MATLAB calculation, this exponent is 0.081. For instance, the time series of x generated from two very close initial values (−1.6,0.82,1.9) and (−1.601,0.82,1.9) are shown in Figure 3, with the purpose of verifying the initial value sensitivity for the chaotic system. Figure 3 is plotted by the MATLAB numerical simulation. According to the Differential Equation (1), the “*t*” presents the number of iterations. As can be seen from Figure 3, the chaotic system is sensitive dependence on initial value.

## 3. A New Algorithm to Improve the Complexity of Digital Chaotic Sequences

In this section, we designed a novel algorithm based on CEEMD that can effectively enhance the complexity of digital chaotic sequences. CEEMD can adaptively decompose a non-stationary or non-linear signal into different IMFs. The oscillating frequency of each IMF decreases according to the decomposition order of each IMF. We present a new algorithm to enhance the complexity of chaotic discrete sequences by combining CEEMD with permutation entropy (PE). At the same time, the digital chaotic sequences are converted into chaotic binary sequences through a quantitative method with the purpose of encrypting images or private data. The essential novelty of this algorithm is to eliminate all low complexity IMF components in a chaotic time series, with the purpose of improving the randomness and complexity of the sequence.

### 3.1. The Basic Principles of EMD

Empirical mode decomposition (EMD) is an adaptive method to decompose non-stationary and non-linear signals into a set of IMFs (intrinsic mode functions) and a residual component. Each IMF should satisfy the following two conditions: (1) For the whole data set, the number of zero crossing and extrema must either be equal or differ at most by one. (2) For any data point, the mean value of the upper and lower envelope determined by the local maxima and minima is zero [31]. The implementation process of the EMD method is shown as follows:

1. All the local maxima and minima of the signal s(t) are calculated to construct the upper envelopes $e_{+}(t)$ and lower envelopes $e_{-}(t)$ by the cubic spline interpolation. Further, $m_{11}(t)$ represents the mean of the upper and lower envelopes and is shown as follows:
  (3) $$m_{11}(t)=\frac{e_{+}(t)+e_{-}(t)}{2}$$
  (4) $$s(t)-m_{11}(t)=h_{11}(t)$$
  where $h_{11}(t)$ denotes a temporary signal. If $h_{11}(t)$ satisfies the above two crucial factors, it is a first-order IMF component. Otherwise, $h_{11}(t)$ will serve as an initial signal and the above procedures are repeated until the $h_{1k}(t)$ is an IMF and sets the $h_{1k}(t)$ as $c_{1}(t)$.
  (5) $$c_{1}(t)=h_{1k}(t)$$
2. Next, the first-order IMF has a high frequency, which can be extracted from s(t) by
  (6) $$s(t)-c_{1}(t)=R_{1}(t)$$
  $R_{1}(t)$ is processed as the new signal and the above procedures are repeated so that the other IMFs can be generated $R_{i}(t)$, i=2,⋯,n.
3. When the residual $R_{n}(t)$ becomes a monotonic function or constant, EMD decomposition is terminated. The s(t) can finally be shown as follows:
  (7) $$s(t)=\sum_{i=1}^{n}c_{i}(t)+R_{n}(t)$$
  Thus, a non-linear signal s(t) can be decomposed into n IMFs and a residual $R_{n}(t)$. However, there are some problems with the EMD method, and one of these is mode mixing. Generally speaking, each IMF component represents a specific physical quantity. If an IMF component contains a large number of different frequencies of signals then this phenomenon is called mode mixing, which seriously affects the performance of EMD decomposition. Aiming to resolve this issue, the complementary ensemble empirical mode decomposition (CEEMD) method can effectively restrain mode mixing of EMD at a certain level. The CEEMD method was used by adding two opposite white noise signals to an original signal s(t), and to the adopted EMD, with the purpose of restraining mode mixing.

### 3.2. The Implementation of the New Algorithm

First, suppose x(t) is a time series of chaotic systems. The white noise signal $w_{i}(t)$ and $-w_{i}(t)$ with a zero mean value are added to the signal x(t), and the following equation is defined:

(8) $$\{\begin{array}{cc} \begin{array}{c} x_{i}^{+}(t)=x(t)+a_{i}w_{i}(t)\\ x_{i}^{-}(t)=x(t)-a_{i}w_{i}(t) \end{array} & 1\leq i\leq N_{p} \end{array},$$

where $w_{i}(t)$ shows the added white noise signal, and $a_{i}$ and $N_{p}$ denote the amplitude and number of the noise signals, respectively. In addition, the variance of the white noise is 1. $\left\{I_{1i}^{+}(t)\right\}$ and $\left\{I_{1i}^{-}(t)\right\}$ $\left(1\leq i\leq N_{p}\right)$ represent the first order component sequence, which can be generated by decomposing $x_{i}^{+}(t)$ and $x_{i}^{-}(t)$ with the EMD method. The mean value of all components is defined as follows:

(9) $$I_{1}(t)=\frac{1}{2N}\sum_{i=1}^{N_{p}}\left[I_{1i}^{+}(t)+I_{1i}^{-}(t)\right].$$

$I_{1}(t)$ is sampled to generate a discrete time sequence $I_{1}(n)$. Then, it is checked whether $I_{1}(n)$ is a low complexity discrete sequence based on the PE value. The PE is widely applied in the measurement of discrete sequence complexity because of its high robustness and rapid and simple algorithm characteristics. PE can be described as follows:

1. For a discrete time sequence $X_{N}=\left\{\begin{array}{cccc} X_{1}, & X_{2}, & \cdots & X_{N} \end{array}\right\}$, where m and τ represent the embedding dimension and a delay factor, respectively, the sequence $X_{N}$ can be reconstructed as

(10) $$\begin{array}{cc} X(n),X(n+\tau ),\cdots ,X(n+(m-1)\tau ) & 1\leq n\leq N-m+1 \end{array},$$

2. Each sequence of Equation (10) is placed depending on an ascending order.

(11) $$X(n+(k_{1}-1)\tau )\leq X(n+(k_{2}-1)\tau )\leq \cdots \leq X(n+(k_{m}-1)\tau ),$$

3. Further, $\pi_{n}=\left\{\begin{array}{cccc} k_{1}, & k_{2}, & \cdots , & k_{m} \end{array}\right\}$ displays the original position index of each element, which is one of the possible order types of all m! permutations. Suppose $P_{g}$ is a symbol permutation and $\sum_{g=1}^{w}P_{g}=1$, where g=1,2,⋯,w, w≤m!. Then, PE $H_{p}$ is defined as

(12) $$H_{p}=-\sum_{g=1}^{w}P_{g}\ln P_{g}.$$

When $H_{p}=1/m!$, then $H_{p}$ obtains the maximum value ln(m!). Further, the normalized PE $h_{p}$ is defined as $h_{p}=H_{p}/\ln(m!)$.

Based on a large amount of MATLAB simulation data, when the PE value of the $I_{1}(n)$ is less than θ∈[0.5,0.6], the amplitude of $I_{1}(n)$ changes slowly and takes on a lower frequency. After this, the above method is used to find all the low complexity signals in the IMFs. All low complexity IMF signals are separated from the target signal x(t) to generate the signal r(t). Then, the r(t) can be written as

(13) $$r(t)=x(t)-\sum_{j=1}^{p}I_{j}(t).$$

where p is the sum total of low complexity signals in the IMFs.

### 3.3. Experimental Results

**Figure 4:**
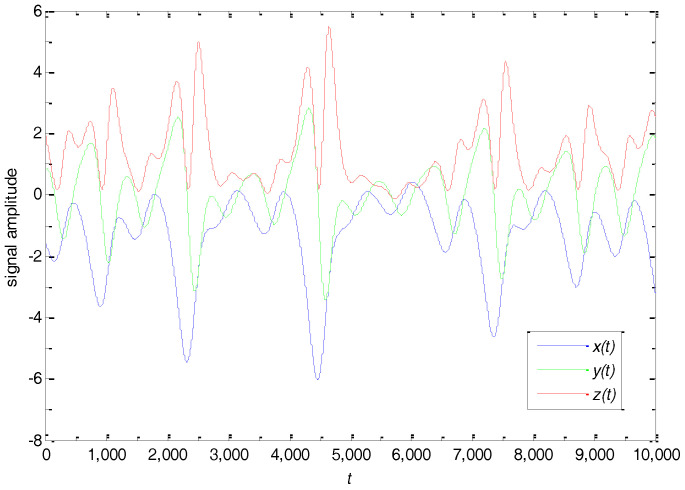
Chaotic time series with x(t) (blue color), y(t) (green color), and z(t) (red color).

The time series (x(t),y(t),z(t)) are generated from the chaotic system as experimental data. The generated x(t), y(t) and z(t) time series signals are shown in Figure 4.

**Figure 5:**
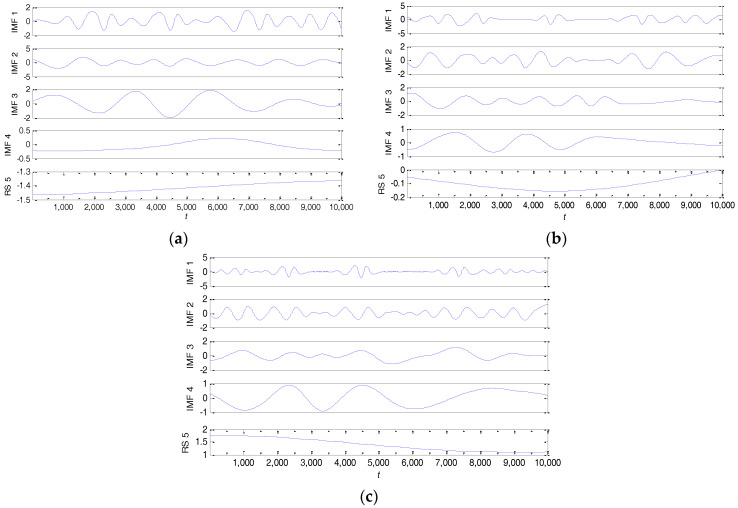
All the low complexity signals in the intrinsic mode functions (IMFs) with: (**a**) x(t); (**b**) y(t); (**c**) z(t).

Next, these chaotic time series are processed by the above method. All the low complexity signals in the IMFs are shown in Figure 5a–c, where RS (Logogram of Residual $R_{n}(t)$) is a residual signal. As can be seen from the figure, the amplitude of these IMF signals changes slowly with time and the frequency of the signals reduces. These IMF components are sampled to generate discrete time sequences with the purpose of calculating the PE value. For the x(t), y(t) and z(t) time series, the calculation results of the PE value of each IMF component are shown in Table 1. This table shows that the PE values of these IMFs are less than θ∈[0.5,0.6]. Therefore, based on the essential novelty of the above method, these IMFs will be removed from the original chaotic time series.

**Figure 6:**
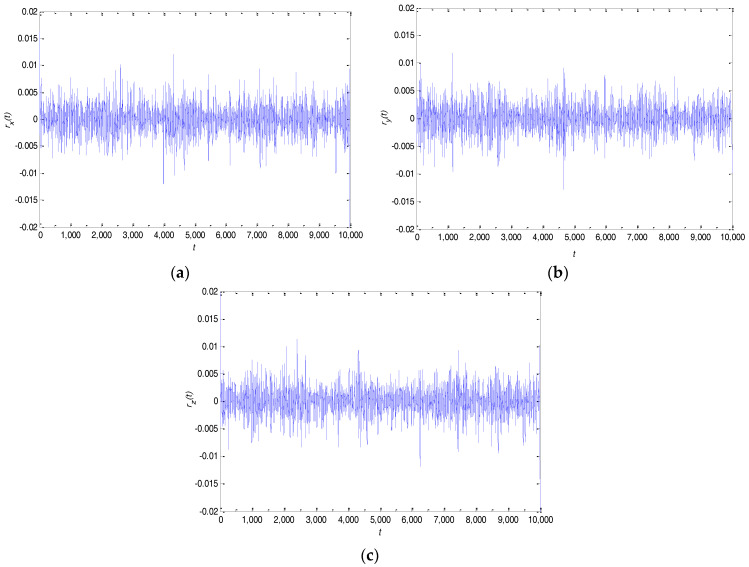
Time series after algorithm processing with: (**a**) x(t); (**b**) y(t); (**c**) z(t).

**Figure 7:**
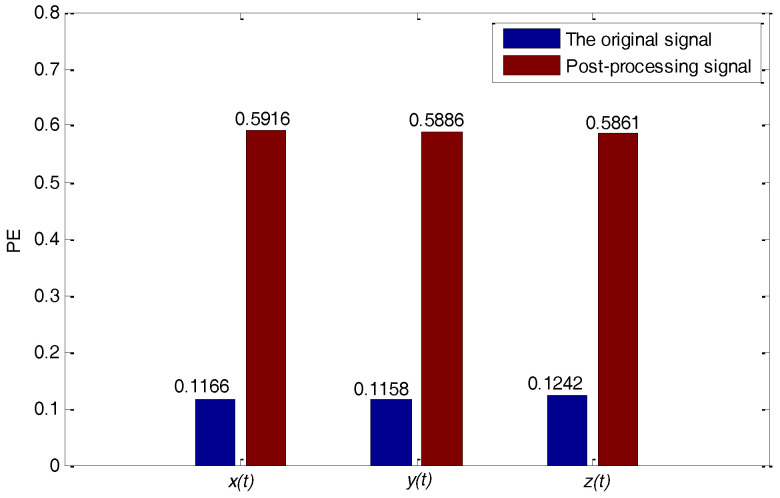
Permutation entropy (PE) value comparisons between the original signal and post-processing signal.

The time series $r_{x}(t)$, $r_{y}(t)$ and $r_{z}(t)$ will be generated by removing the low complexity IMF components from the original signals in x(t), y(t) and z(t). The time series $r_{x}(t)$, $r_{y}(t)$ and $r_{z}(t)$ are shown in Figure 6. Moreover, these time series are also sampled to generate discrete time sequences with the purpose of calculating the PE values, and Figure 7 denotes the comparison of the PE values to the original signals x(t), y(t), z(t) and the post-processing signals $r_{x}(t)$, $r_{y}(t)$, $r_{z}(t)$. It can be seen from Figure 7 that the entropy value of the latter is significantly greater than that of the former and shows a good level of complexity. These high-complexity discrete time sequences can be quantized to generate a good performance in the chaotic binary sequences. These binary sequences will serve as useful key stream sequences of the stream cipher to encrypt private data.

### 3.4. The Generation and Performance Test of the Chaotic Binary Sequence

The three outputs $r_{x}(t)$, $r_{y}(t)$, and $r_{z}(t)$ are quantized by the interval quantization method, and its mathematical equation is shown below.

(14) $$\begin{array}{cc} Q_{0-1}(t)=\{\begin{array}{c} \begin{array}{cc} 1, & x(t)\in \overset{2^{m}-1}{\underset{k=0}{\cup }}D_{2k}^{m} \end{array}\\ \begin{array}{cc} 0, & x(t)\in \overset{2^{m}-1}{\underset{k=0}{\cup }}D_{2k+1}^{m} \end{array} \end{array}; & k=0,1,2,\cdots \end{array},$$

where $Q_{0-1}(t)$ and m are a quantized chaotic binary sequence and arbitrary integer, and $D_{0}^{m},D_{1}^{m},D_{2}^{m}\cdots$ are $2^{m}$ consecutive equal intervals on the range of the real value of x(t). If the real value falls on the odd range the result of quantization is 0, otherwise it is 1. $r_{x}(t)$, $r_{y}(t)$, and $r_{z}(t)$ are quantized as $Q_{x}(t)$, $Q_{y}(t)$, and $Q_{z}(t)$ through the interval quantization method. Then, the NIST-800-22 test suite is performed to evaluate the performance of the random binary sequences $Q_{x}(t)$, $Q_{y}(t)$, and $Q_{z}(t)$. The NIST-800-22 is composed of 16 different tests, including approximate entropy, linear complexity, and the discrete Fourier transform tests [32,33]. If the *p*-value of the test is greater than 0.01, the test is successful. The NIST-800-22 test results are shown in Table 2. As can be seen from the table, the chaotic random sequences $Q_{x}(t)$, $Q_{y}(t)$, and $Q_{z}(t)$ passed all the tests. These chaotic sequences can be used in high security fields such as network security and multimedia encryption.

## 4. Image Encryption with a Chaotic Binary Sequence

This subsection describes the experiments used to demonstrate the performance of the chaotic binary sequence by encrypting images. The Lena and Baboon images, with a size of 256×256, are encrypted by the above chaotic random sequences—$Q_{x}(t)$, $Q_{y}(t)$, and $Q_{z}(t)$. Then, $Q_{x}(t)$, $Q_{y}(t)$, and $Q_{z}(t)$ serve as the key stream sequences of the stream cipher with the purpose of encrypting the R, G, and B components of the color images.

### 4.1. Key Sensitivity

**Figure 8:**
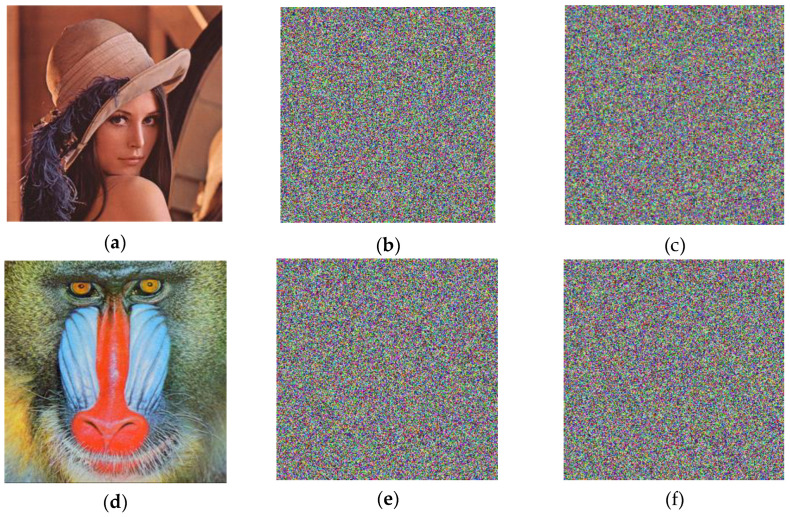
Key sensitivity test with: (**a**) plain-image for Lena; (**b**) cipher-image for Lena; (**c**) incorrect decryption using a 10^−5^ change of the initial value for Lena; (**d**) plain-image for Baboon; (**e**) cipher-image for Baboon; (**f**) incorrect decryption using a 10^−5^ change of the initial value for Baboon.

**Figure 9:**
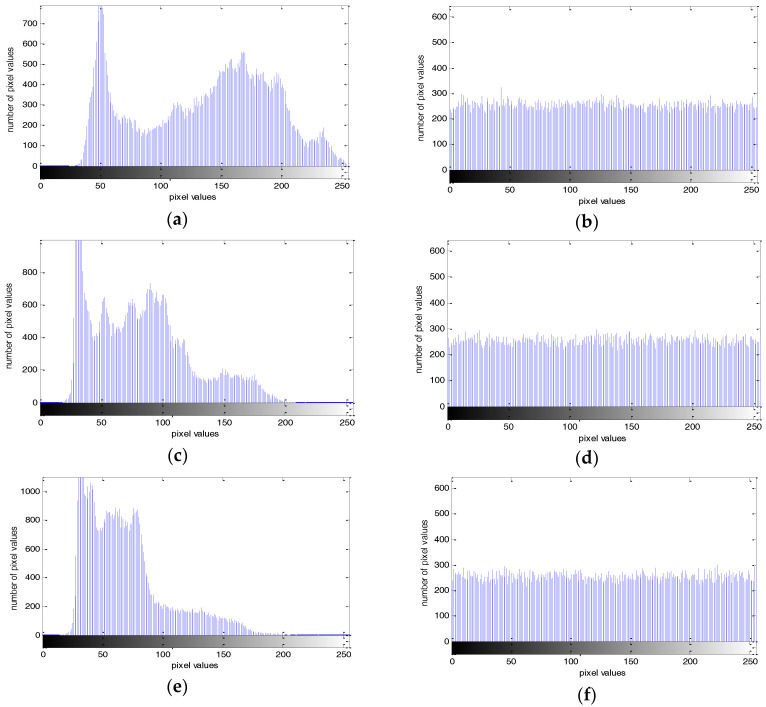
Histogram test with: (**a**) R component of the plain-image; (**b**) R component of the cipher-image; (**c**) B component of the plain-image; (**d**) B component of the cipher-image; (**e**) G component of the plain-image; (**f**) G component of the cipher-image.

The sensitivity of chaos to the initial value can support the effective avoidance of tentative attacks. Using the Lena and Baboon images with a size of 256×256 as examples, Figure 8a,d shows the plain-images, while the cipher-images are given in Figure 9b,e. However, a 10^−5^ change of the initial value will lead to incorrect decryption results, as shown in Figure 9c,f. The experimental results show that the chaotic binary sequence shows high key sensitivity.

### 4.2. Histogram Analysis

The image histogram can be approximated as the density function of the gray value, which is an important indicator in the analysis of an image’s statistical properties [34]. The histogram test is shown in Figure 9, and the horizontal and vertical coordinates of the histogram represent the pixel values and number of pixel values, respectively. Figure 9 show that the gray histogram of the encrypted image is relatively uniform, which indicates that the security performance of this key sequence is relatively high, and the image is not easily able to be tampered with and decrypted during transmission.

### 4.3. Correlation Analysis of Adjacent Pixels

Generally speaking, the smaller the adjacent pixel correlation of the cipher-image, the more obvious the effect of resisting statistical attack [35]. The mathematical equation can be shown as follows:

(15) $$\rho_{xy}=\frac{\mathrm{cov}(x,y)}{\sqrt{D(x)D(y)}}.$$

where $\mathrm{cov}(x,y)=\frac{1}{N}\sum_{i=1}^{N}\left(x_{i}-E(x))(y_{i}-E(y)\right)$, $D(x)=\frac{1}{N}\sum_{i=1}^{N}{\left(x_{i}-E(x)\right)}^{2}$, $E(x)=\frac{1}{N}\sum_{i=1}^{N}x_{i}$, *x_i_* and *y_i_* represent the different gray values of two adjacent pixels and *N* denotes the number of randomly selected adjacent pixels.

The above equation was used and some pairs of adjacent pixels in different directions were randomly chosen, and the test results are listed in Table 3. It can be seen from the experimental data that the correlation of adjacent pixels of a cipher-image tends to be zero.

### 4.4. Information Entropy Analysis

Information entropy can reflect the randomness of the information in images, namely the uncertainty of the distribution of pixel values in a cipher-image. Its mathematical equation is shown below [36].

(16) $$H(\phi )=\sum_{i=0}^{2^{L}-1}p(\phi_{i})\log_{2}\frac{1}{p(\phi_{i})}.$$

where *L* is the number of bits required to store each pixel value, and *p*(*ϕ_i_*) presents the probability of the symbol *ϕ_i_*. When the probability of each symbol *ϕ_i_* is equal, the information entropy (*H*(*ϕ*) = 8) is at its largest. When the information entropy is closer to 8, the gray value tends to be distributed randomly. Table 4 provides a comparison of this data with other experiments. This comparison shows that the information entropy of our method is closer to 8. Therefore, it can effectively resist information entropy attacks.

## 5. Discussion

Some traditional chaotic systems with a self-excited attractor have been widely used in secret communication. However, for chaotic systems with hidden attractors, most of the current research has focused on studying the dynamic characteristics of the system rather than its application in the field of information security. Therefore, in this paper, we aimed to study chaos with a hidden attractor from the perspective of secure communication and data encryption. First, we introduced the dynamic characteristics of a chaotic system with hidden attractors by means of a numerical simulation and theoretical analysis, including equilibria, a Poincaré cross section, and initial value sensitivity. After that, a new algorithm was designed to enhance the complexity of digital chaotic sequences with the purpose of satisfying the requirements of data encryption. The essential novelty of the algorithm is to eliminate all low complexity IMF components of a chaotic time series by using digital signal processing technology. PE value comparisons between the original signal and post-processing signal show the performance of the algorithm is good. In addition, the NIST-800-22 test was performed to demonstrate the randomness and complexity of the chaotic binary sequence. The chaotic binary sequence can serve as a good key stream sequence of a stream cipher to encrypt private data. Furthermore, an image encryption experiment was undertaken to show the security of the above method. However, some weaknesses in this technique remain, and we believe that the new algorithm should be optimized in operation efficiency.

## Figures and Tables

**Table 1:** The permutation entropy (PE) value of each intrinsic mode function (IMF) with x(t), y(t), and z(t).

IMF Component	x(t)	y(t)	z(t)
IMF1	0.1181	0.1959	0.1658
IMF2	0.1116	0.1153	0.1198
IMF3	0.1096	0.1113	0.1102
IMF4	0.1069	0.1076	0.1072
RS5	0.0542	0.0997	0.1066

**Table 2:** NIST-800-22 tests.

Test Item	$Q_{x}(t)$*p*-Value	$Q_{y}(t)$*p*-Value	$Q_{z}(t)$*p*-Value	Result
Approximate Entropy	0.28711	0.01063	0.41042	Success
Block Frequency	0.02501	0.43924	0.64085	Success
Cumulative Sums	0.14372	0.56658	0.64761	Success
FFT	0.52063	0.37221	0.11875	Success
Frequency	0.28014	0.48392	0.87461	Success
Linear Complexity	0.22374	0.46932	0.78321	Success
Longest Run	0.70665	0.51078	0.26541	Success
Non-Overlapping Template	0.32974	0.75331	0.11253	Success
Overlapping Template	0.24088	0.70399	0.32227	Success
Random Excursions	0.43747	0.51791	0.82733	Success
Random Excursions Variant	0.64578	0.11253	0.66691	Success
Binary Matrix Rank	0.15319	0.58700	0.44130	Success
Runs	0.88206	0.84530	0.71884	Success
Serial Test-1	0.10056	0.17826	0.81473	Success
Serial Test-2	0.15538	0.15538	0.69926	Success
Maurer’s Universal	0.75331	0.14268	0.56553	Success

**Table 3:** Correlation analysis of adjacent pixels for the Lena and Baboon images.

Direction	Plain-Image for Lena	Cipher-Image for Lena	Plain-Image for Baboon	Cipher-Image for Baboon
Horizontal	0.9712	0.0392	0.9287	0.0133
Vertical	0.9655	0.0091	0.9004	0.0522
Diagonal	0.9401	0.0215	0.8711	0.0093

**Table 4:** Information entropy analysis for the Lena and Baboon images.

Methods	R Component	G Component	B Component
The paper for Lena	7.9972	7.9971	7.9972
The paper for Baboon	7.9970	7.9968	7.9971
Reference [37]	7.9914	7.9914	7.9915
Reference [38]	7.9851	7.9852	7.9832
